# Process Evaluation Tool Development and Fidelity of Healthy Retail Interventions in American Indian Tribally Owned Convenience Stores: the Tribal Health Resilience in Vulnerable Environments (THRIVE) Study

**DOI:** 10.1093/cdn/nzz073

**Published:** 2019-06-25

**Authors:** Tori E Taniguchi, Alicia L Salvatore, Mary B Williams, Charlotte V Love, Carolyn J Noonan, Tamela K Cannady, Joy Standridge, Jill Fox, Jennifer Spiegel, JoAnna Owens, Mandy Grammar, AnDina Wiley, Valarie Blue Bird Jernigan

**Affiliations:** 1 Center for Indigenous Health Research and Policy, Oklahoma State University Center for Health Sciences, Tulsa, OK, USA; 2 Department of Health Promotion Sciences, University of Oklahoma Health Sciences Center College of Public Health, Oklahoma City, OK, USA; 3 Department of Biostatistics and Epidemiology, University of Oklahoma Health Sciences Center College of Public Health, Oklahoma City, OK, USA; 4 Initiative for Research and Education to Advance Community Health, Washington State University, Pullman, WA, USA; 5 Choctaw Nation of Oklahoma Health Services Authority, Durant, OK, USA; 6 Chickasaw Nation Nutrition Services Department, Purcell, OK, USA

**Keywords:** process evaluation, obesity, American Indians, healthy retail intervention, food environment, tribal convenience store

## Abstract

**Background:**

The Tribal Health Resilience in Vulnerable Environments (THRIVE) study aimed to increase healthy food access in 2 rural American Indian communities. The intervention sought to increase fruit and vegetable availability, variety, and convenience through placement, promotion, and pricing of healthy foods and beverages in tribal convenience stores.

**Objective:**

The aim of this study was to describe the development and implementation of the study process evaluation tool to assess intervention fidelity as part of this cluster-controlled trial.

**Methods:**

Eight stores (2 intervention and 2 control stores per Nation) participated in the study, implemented from May 2016 to May 2017. A web-based survey tailored to store layouts and intervention components assessed how often intervention items were available, approximate quantity available, and whether placement of healthier food items and promotional materials were implemented as designed. After pilot testing the survey, tribal staff members implemented it to collect process evaluation data in the 8 stores during a period of 9–12 mo, assessing study implementation and potential changes in control stores.

**Results:**

Promotional materials were available ≥75% of the time for most intervention locations. Fruit availability was similar in Nation A and Nation B intervention stores (79–100% compared with 70–100%), whereas fresh vegetable availability was higher in Nation B compared with Nation A (95–96% compared with 55–75%). Both control stores in Nation A and 1 control store in Nation B had moderate fruit and vegetable availability, ranging from 45% to 52%. No control stores in either Nation used intervention promotional materials.

**Conclusions:**

Process evaluation data indicate that the study was implemented with moderate to high fidelity. The development and implementation of the tool can inform future healthy retail interventions that aim to improve rural and tribal food environments.

## Introduction

American Indians (AIs) have higher rates of obesity, hypertension, and diabetes compared with the US general population (1). In rural Oklahoma, AIs experience significant diet-related disparities (2) and have limited access to healthy, fresh foods (3). AI households also experience disproportionally higher food insecurity (30%) compared with non-AI households (14%) (3). Food insecurity, defined as the limited availability of nutritional and safe foods (4), is associated with poor food environments, which are common in rural AI communities (5, 6). Research has indicated that interventions that increase access to healthy foods are key to addressing obesity and other diet-related health disparities in communities with poor food environments (7).

Healthy retail interventions that improve the quality, availability, and affordability of fruits, vegetables, and other healthy foods in existing grocery, corner, and convenience stores have gained prominence during the past decade (8). Although healthy retail interventions have been conducted with other populations, few have focused on AI communities. Only 2 studies have examined the efficacy of healthy retail interventions in AI communities. These studies implemented cooking demonstrations and taste tests in grocery stores in First Nations, Inuit, and AI communities in the southwestern United States (9–11). The interventions increased knowledge and frequency of healthy food purchasing; 1 of the studies reported reductions in BMI among participants who shopped most frequently at the stores (9). At the store level, 1 of the studies achieved a moderate to high fidelity in terms of promoted food availability, appropriate shelf labeling, and the presence of posters and educational displays (10). The studies cited a lack of participation by convenience stores as well as a lack of engagement with tribal leaders and policymakers as study limitations (9).

Convenience stores—defined by the Association for Convenience and Fuel Retailing as retail businesses that provide a convenient location to quickly purchase a wide array of consumable products, predominantly food or food and gasoline and services (12)—are an important economic opportunity for sovereign Tribal Nations. In 2014, there were 180 tribes with 293 tribally owned convenience stores in 25 states, and this number continues to increase (13).

The Tribal Health Resilience in Vulnerable Environments (THRIVE) study is a 5-y community-based participatory research (CBPR) study aimed to improve the tribal food environments by implementing healthy retail interventions in tribally owned convenience stores in the Chickasaw Nation and Choctaw Nation of Oklahoma (14). To our knowledge, this was the first healthy retail intervention to be implemented in tribally owned convenience stores (14). We describe the process evaluation tool developed to track intervention fidelity and the results of these evaluations to assess fidelity throughout the THRIVE intervention period.

## Methods

### Setting and context

The Chickasaw Nation and Choctaw Nation of Oklahoma are among the largest sovereign indigenous Nations in the United States. Both Tribal Nations are located in the southeastern area of Oklahoma and are similar in size, population, and rurality. Together, their land mass comprises one-fourth of the state of Oklahoma, and they have a combined population of nearly 300,000 citizens (2). Poverty rates for all residents of the Chickasaw Nation and Choctaw Nation of Oklahoma are 15.3% and 20.7%, respectively, compared with the national poverty level of 13.8% (15). The Chickasaw Nation and Choctaw Nation of Oklahoma own more than 6 convenience stores each. Tribally owned convenience stores represent a unique opportunity as a suitable setting for healthy food retail interventions (16). The tribal convenience stores in these Nations are similar to non-tribal convenience stores in size, scope, and products sold. The stores are an important source of revenue for both Nations and are frequented by both Native and non-Native residents. Data from our first survey of 513 AIs living in the Chickasaw Nation and Choctaw Nation of Oklahoma revealed that more than 60% of those surveyed reported purchasing food at tribally owned convenience stores at least 3 times per week (3).

### THRIVE intervention and trial design

The THRIVE intervention was designed to increase fruit and vegetable availability, variety, and convenience through placement, promotion, and pricing of healthy foods throughout 4 tribally owned convenience stores (14). Our tribal–university partnership, including tribal leaders from the health, commerce, government, and research divisions of both Nations and public health researchers and dietitians at the University of Oklahoma, adapted and localized evidence-based healthy food retail strategies recommended by the Institute of Medicine and CDC (17). Both the participatory research processes (14) and outcomes (18) of the THRIVE study are published elsewhere. The THRIVE study was approved by the University of Oklahoma Health Sciences Center, the Chickasaw Nation, and the Choctaw Nation of Oklahoma institutional review boards (IRBs). Per request of the Chickasaw Nation and Choctaw Nation of Oklahoma IRBs, the Nations are not named when describing study data.

Tribal leaders from the health, commerce, government, and research divisions of each Nation, in collaboration with university researchers and dietitians, identified ≥11 fruit and vegetable items, including fresh and canned, and 5 healthier meals, including salads, and procured a minimum of 20 healthy snack items to introduce or promote. Food vendor catalogs were also reviewed by tribal community nutrition researchers to identify the specific types of healthy foods that could be obtained by tribal convenience stores (19). Healthy snacks and meals were chosen based on guidelines of total calories and percentage of calories from fat recommended in the Nutrition Environment Measurement Survey in Stores (NEMS-S) (20). More information about the development of the tool for the intervention is provided elsewhere (19).

Placement strategies, carried out by store managers, included placing large, open-air coolers in the center of the stores stocked with the healthier options. Store managers in each Nation also displayed fresh fruit in baskets near the intervention store entrances and used other prominent locations, including endcaps (i.e., the end of the aisles) and areas near the cash registers. The promotion strategies entailed the marketing of healthy foods with in-store signs and displays, including promotional signage in Native language. Both Nations offered combination meals priced at 30% below the sum of the individually priced items to compete with the most popular less healthy combination meals. Provision of marketing tools and introduction of healthy foods into the intervention stores occurred 1 mo before the trial began. Tribal researchers were responsible for ensuring products and promotional materials were placed as intended.

Four stores from each Nation participated in the trial, with 2 receiving the intervention and 2 serving as control stores. The initial intervention was planned for 6 mo in both Nations but was extended for 9 mo in Nation A stores and 12 mo in Nation B stores. The THRIVE study assessed both individual- and store-level effects using a cluster-controlled trial with treatment condition allocated at the store level. More information on the individual- and store-level effects of the THRIVE intervention is presented elsewhere (18, 19).

### Process evaluation tool and measures

Because this intervention was implemented as a cluster-control study at the store level, the process evaluation tool was designed to measure implementation fidelity and the extent to which specific intervention strategies and intervention components were present in control stores during the intervention study.

**FIGURE 1:**
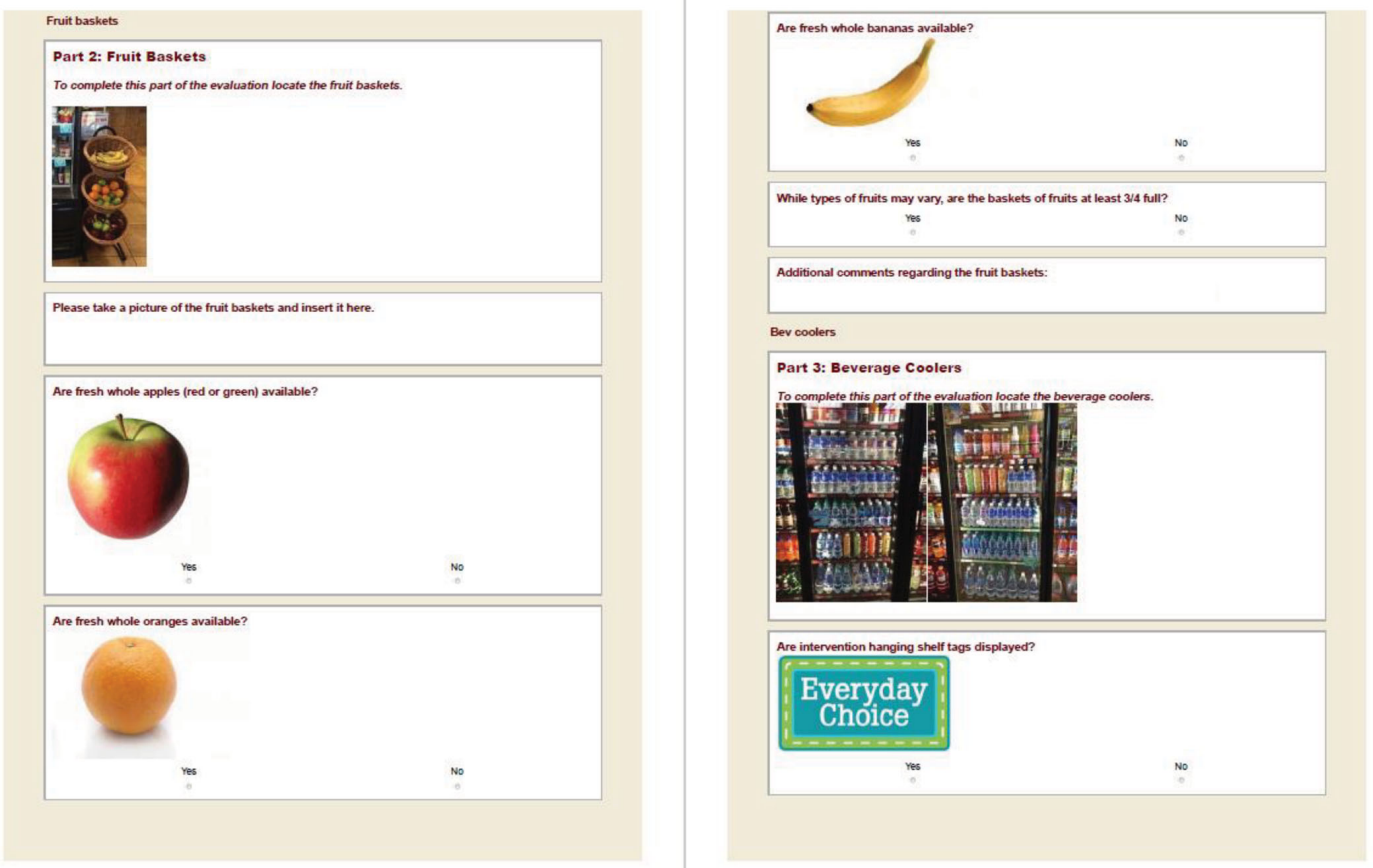
Sample screens from the THRIVE process evaluation tool.

Guided by tribal partners and tribal economic leaders, we developed a detailed, visual, intervention-specific, web-based tool (**Figure 1**) for the evaluation. The tool measured whether intervention items were regularly available, the approximate quantity available, and whether placement and promotional elements of the intervention were implemented as planned, including the availability and placement of healthier food items (e.g., fruits, vegetables, and other healthy foods and beverages) and promotional materials (e.g., store shelf labels, signs, tags, and displays) (**Table 1**). Because we used a CBPR approach for the intervention, we endorsed tribal partners and tribal economic leaders from both Nations to decide which specific intervention items they wanted to focus on promoting in their stores. Based on final decisions of intervention products to promote, similar tailored versions of the tool were created for each Nation to reflect the different intervention strategies selected and the distinct store layouts. For example, the tool version for Nation A assessed the availability of any fresh fruit cups, given variations in types of cups by vendor, whereas in Nation B, the tool assessed the specific availability of blueberry/strawberry cups, grape cups, strawberry cups, and fruit-blend cups. Also, the tool in Nation B differed slightly by store. For example, due to vendor differences, 1 store in Nation B promoted different snacks compared with the other stores.

**TABLE 1:** Healthy retail strategies implemented in tribally owned convenience stores, THRIVE intervention^1^

Evidence-based strategy	Specific strategies implemented
Products	Increased availability, variety, and convenience of healthier products^2^ (including at least 10 new snack choices and 5 new meal choices)
	Packaged vegetable and fruit “quick packs”
	Nation B expanded kitchens; prepared and served healthier food items^2^ “in house”
Placement	Purchased and placed open-air coolers at storefront entrances Relocated fried food hot boxes behind registers
	“Rented” endcap space and stocked with healthier items^2^
Promotion	Labeled healthier foods and drinks on shelves and coolers (e.g., “quick and healthy,” “everyday choice,” “better choice,” and “fresher option”)
	Placed promotional signage above coolers (e.g., “Fresh Food Destination” and “Good and Good for You”)
Pricing	Offered discounted healthier meal combos^2^ (including meal, snack, and bottled water)
	Priced healthier meals and snacks at or below prices of competing foods

1 THRIVE, Tribal Health Resilience in Vulnerable Environments.

2 Guided by NEMS criteria: <500 calories and 30% or fewer calories from fat for meals; <200 calories and 35% or fewer calories from fat for snacks (excluding nuts/seeds).

The tool was implemented by tribal researchers who participated in a 1-d training session led by university study investigators. The morning session included didactic instructions on how to use the web-based tool, protocols for documenting and addressing various scenarios (i.e., what to do when products were not available, placement was incorrect, or promotion was absent), and guidance on completing qualitative notes where feasible within the tool. The afternoon session of the training focused on practicing using the tool within several of the store environments and technical assistance to address any issues that arose.

The specific process evaluation items measured were mapped to intervention store locations and are discussed next.

#### Promotional materials

In both Tribal Nations, process evaluation items measured whether signs and tags to promote intervention foods and drinks were displayed on reach-in food coolers, endcaps, grocery shelves, and beverage coolers. In Nation A, we included items to assess the presence of channel strips on the reach-in food cooler and endcaps and promotional signage on the cereal/oatmeal stands. In Nation B, we included items to assess the presence of signage on fruit baskets, beverage fountains, and gas pumps.

#### Fruits and vegetables

In both Tribal Nations, process evaluation items measured the presence of fresh fruit, packaged fresh vegetables, 100% vegetable juice, and salads in the reach-in food coolers and fruit baskets. In Nation A, process evaluation items also measured the presence of canned fruit in the reach-in food cooler or in the grocery shelves. In Nation B, process evaluation measures included items to assess whether 100% vegetable juice was promoted and present in the beverage cooler.

#### Other healthy foods and beverages

In both Tribal Nations, process evaluation items measured the presence of other healthy foods and beverages, including healthy snacks, plain cereal/oatmeal, canned lean meats/fish, and bottled water located in the reach-in food cooler, endcaps, cereal/oatmeal stand (Nation A only), and beverage cooler. In Nation B, bottled water was present and promoted in the reach-in food cooler and the beverage cooler, whereas in Nation A, bottled water was only present and promoted in the beverage cooler. In Nation B, process evaluation items also measured the presence of healthy sandwiches and wraps, hummus packs, and milk/milk substitutes as part of the intervention located in the reach-in cooler.

The process evaluation tools were piloted and refined by our tribal–university research team. Tribal staff who were not involved in the implementation of the intervention activities used this tool on electronic tablets to collect routine process evaluation data in the 8 convenience stores.

### Intervention fidelity

Fidelity was evaluated by capturing the availability and proper placement of promotional materials and availability of intervention food items (e.g., fruits, vegetables, and other healthy foods and beverages). Evaluations were conducted in both intervention and control stores to assess the implementation of the THRIVE interventions as well as potential changes in control stores. Process evaluations were collected for the entire 9-mo intervention period in Nation A. In Nation A, process evaluation assessments in the intervention stores were conducted once every week for the first 2 mo, with some gaps of once every 2 wk due to variations in staff availability. For the remaining 7 mo, intervention stores were evaluated once every 2 wk. The control stores in Nation A were evaluated once every 2 wk for the entire 9 mo. Due to staff limitations, process evaluations were collected for 10 mo in Nation B. In Nation B, the intervention stores were evaluated once every week for the first 2 mo, once every 2 wk for the next 4 mo, and once per month for the remaining months. In Nation B, control stores were evaluated once every 2 wk for 6 mo and then once per month for the remaining 4 mo.

### Data management and analysis

We assessed intervention fidelity by calculating the percentage of evaluations reporting adherence to the intervention design for promotional material displays and product availability. Adherence to the intervention for promotional materials was defined as displaying at least 1 promotional item in each target location (e.g., reach-in food coolers, endcaps, grocery shelves, and beverage coolers) on the report, and product availability intervention adherence was defined as the availability of at least 1 item in the food category in the target location on the evaluation. **Supplemental Tables 1** and **2** list the number of individual promotional materials and food items that were collapsed for each category for analyses. When there were multiple food items for a category (e.g., multiple flavors of nutrition bars), we first calculated the percentage of evaluations reporting adherence for product availability for each item and then averaged the percentages across items. A few intervention items that were only available in 1 intervention store were not included in analyses (e.g., Skinny Pop popcorn, Harvest Snapea Crisps, and Snack Factory Pretzel Crisps). Fidelity was based on previous process evaluations of healthy eating interventions in Baltimore; low fidelity was considered 0–49%, moderate fidelity was 50–74%, and high fidelity was 75–100% (21). All analyses were conducted with Stata version SE 12.0 (StataCorp) (22).

## Results

### Process evaluation tool

Tribal researchers from both Nations reported that the process evaluation tool was easy to use and protocols were easy to implement as directed in the 1-d training. Challenges that arose during assessments were intervention products that were unavailable due to vendor issues and incorrect placement of promotional materials. These were noted in the process evaluation, and evaluators corresponded with store managers to try to resolve these challenges. For example, on 1 process evaluation, the evaluator mentioned that there were 3 intervention items missing from the reach-in cooler. After speaking with the store manager, the manager verified those items were out of stock but were being ordered. Tribal researchers were able to connect to successfully fill out the survey online. However, in 1 intervention store in Nation A, several process evaluations were completed on paper due to internet connection issues.

### Process evaluation observations

**FIGURE 2:**
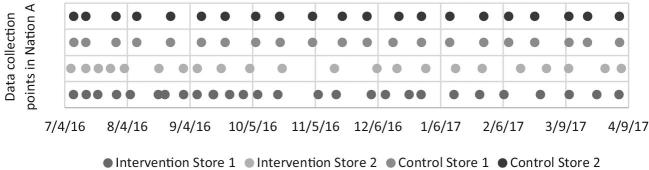
Process evaluation data collection time points in Nation A.

**FIGURE 3:**
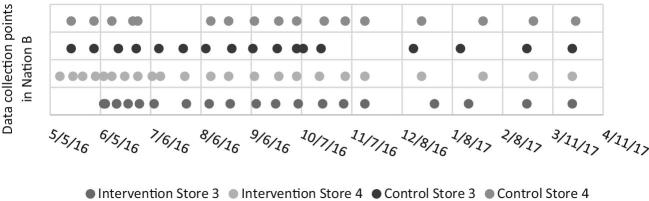
Process evaluation data collection time points in Nation B.

In Nation A, during the 9-mo intervention period from July 2016 to April 2017, 21–28 process evaluation assessments were collected in all 4 stores. In Nation B, during the 12-mo intervention period from May 2016 to May 2017, 17–23 process evaluation assessments were collected in all 4 stores. **Figures 2** and **3** show the process evaluation data collection time points for each store in both Nations. The average number of days between assessments ranged from 19 to 27 d for stores in Nation A and from 16 to 22 d for stores in Nation B.

### Intervention fidelity

#### Promotional materials

Promotional materials were displayed with high fidelity (≥75% of the time) for all but 1 intervention location in Nation A stores and all except 5 intervention locations in Nation B (**Table 2**). In Nation A, there were 4 locations where promotional materials were displayed 100% of the intervention period (endcap signs/tags, endcap channel strips, grocery shelf signs/tags on canned goods, and grocery shelf signs/tags on nutrition bars). Intervention store 1 in Nation A also used channel strips as a promotional material in the reach-in cooler (100% fidelity) and had 96% fidelity in the displaying of promotional signage on the cereal/oatmeal stand. In Nation B, there were 3 locations where promotional materials were displayed 100% of the intervention period (signs/tags in the reach-in cooler, grocery shelf signs/tags on the nutrition bars, and signs on the beverage fountain). In contrast, the fidelity of displaying grocery shelf signs/tags on whole-wheat bread in Nation A (52–56%) and jerky in Nation B (70%) was moderate. Low fidelity of grocery shelf signs/tags on nuts/trail mix (41%) and low-fat/baked chips (9–25%) was observed in Nation B.

**TABLE 2.** Fidelity of displaying THRIVE intervention promotional materials, including signs, tags, and channel strips, in intervention stores^1^

	Nation A		Nation B	
	Intervention store 1, % (*n* = 28)	Intervention store 2, % (*n* = 22)	Intervention store 3, % (*n* = 20)	Intervention store 4, % (*n* = 23)
Reach-in food cooler	82	100	100	100
Endcap	100	100	90	85
Grocery shelf				
Whole-wheat bread	56	52	^2^	^2^
Canned goods	100	100	15	83
Nuts/trail mix	100	86	41	^3^
Nutrition bars	100	100	100	100
Low-fat/baked chips	^2^	^2^	25	9
Pickled quail eggs	^2^	^2^	^4^	87
Jerky	^3^	^3^	70	^3^
Fruit basket	^3^	^3^	100	98
Beverage cooler	100	91	100	67
Beverage fountain	^2^	^2^	100	100
Gas pump	^2^	^2^	95	78

1 THRIVE, Tribal Health Resilience in Vulnerable Environments.

2 Items/locations were not part of the intervention for this Nation; hence, these items/locations were not promoted in these stores.

3 Items in this location were intervention items for this Nation; but items were not promoted in these stores.

4 Items in this Nation were not carried in this store for the entire intervention period; hence, these items were not promoted in the store.

#### Fruits and vegetables

The overall availability of all intervention food and beverage items (fruits, vegetables, other healthy foods, and beverages) ranged from 82% to 93% in all intervention stores in both Nations. The overall availability of fruits and vegetables in intervention stores was high in both Nations, ranging from 88% to 93% (**Table 3**). The availability of promoted fruits in the intervention stores was moderate to high. In Nation A, fresh fruit (fresh fruit cups, fruit and yogurt parfait, apples, oranges, and bananas) availability ranged from 81% to 100% (median: 95.5%) and canned fruit (peaches, mixed fruit, and oranges) ranged from 79% to 100% (median: 100%) (Table 3). In intervention store 1 in Nation A, fruit items that were available 100% of the intervention period were fresh fruit cups, canned mixed fruit, apples, and oranges. In intervention store 2 in Nation A, fruit items that were available 100% of the intervention period were canned peaches, canned mixed fruit, and apples. In Nation B, fresh fruit (fresh fruit cups, sliced apples with caramel/peanut butter, fruit and yogurt parfait, apples, oranges, and bananas) availability ranged from 70% to 100% (median: 92%). In intervention store 3 in Nation B, fresh fruit items that were available 100% of the intervention period were fresh fruit cups, apples, and oranges. Canned fruit was not evaluated as part of the intervention in Nation B. The fidelity of packaged fresh vegetables (95–96% compared with 55–75%) and salads (96–99% compared with 45–76%) was higher in Nation B compared with Nation A, respectively (Table 3). 100% vegetable juice availability was higher in Nation A compared with Nation B (97–100% compared with 73–92%, respectively).

**TABLE 3.** Fidelity of THRIVE intervention product availability by location: fruits and vegetables^1^

	Nation A				Nation B			
	Intervention store 1, % (*n* = 28)	Intervention store 2, % (*n* = 22)	Control store 1, % (*n* = 21)	Control store 2, % (*n* = 21)	Intervention store 3, % (*n* = 20)	Intervention store 4, % (*n* = 23)	Control store 3, % (*n* = 17)	Control store 4, % (*n* = 17)
Reach-in food cooler								
Fresh fruit								
Sliced apples with caramel/peanut butter	^2^	^2^	^2^	^2^	93	84	35	0
Fruit and yogurt parfait	86	91	51	71	90	77	53	0
Fresh fruit cups^3^	100	86	30	100	100	91	82	0
Canned fruit								
Orange fruit cups	86	^4^	0	0	^2^	^2^	^2^	^2^
Peach fruit cups	79	100^5^	46^5^	0	^2^	^2^	^2^	^2^
Mixed fruit cups	100	100^5^	22^5^	0	^2^	^2^	^2^	^2^
Fresh vegetables								
Vegetable packs	75	55	0	22	95	96	35	0
100% juice	100^6^	97^6^	100^6^	17^6^	73	92	17	0
Salads	45	76	34	22	99	96	0	0
Fruit baskets								
Apples	100	100	100	100	100	98	79	0
Oranges	100	95	90	67	100	96	82	0
Bananas	96	81	81	95	85	70	82	0
All fruits and vegetables^7^	88	88	50	45	93	89	52	0

1 THRIVE, Tribal Health Resilience in Vulnerable Environments.

2 These items were not intervention items for this Nation; hence, these items were not evaluated in these stores.

3 Strawberry/blueberry cups, grape cups, strawberry cups, and 4 fruit-blend cups were consolidated in this category for Nation B.

4 Item was not stocked at this store during the intervention period.

5 These items were located in the grocery shelves in these stores.

6 These items were located in the beverage cooler in these stores.

7 Combined fidelity of all fruits and vegetables in reach-in food cooler and fruit baskets.

#### Other healthy foods and beverages

The fidelity of other healthy foods and beverages ranged from moderate to high in both Tribal Nations. The overall availability of other healthy foods and beverages in intervention stores ranged from 73% to 93% in both Nations (**Table 4**). Healthy snack (nutrition bars, jerky, nuts, and trail mix) availability ranged from 56% to 100% (median: 92.5%) in Nation A and from 78% to 100% (median: 90%) in Nation B (Table 4). Canned lean meat/fish (tuna and chicken) availability was high in both Nations, ranging from 96% to 100% in Nation A and from 86% to 100% in Nation B. The fidelity of healthy sandwiches and wraps was high in Nation B, ranging from 84% to –90%. Sandwiches and wraps were not evaluated as part of the intervention in Nation A. The availability of bottled water at all 4 intervention stores was 100%, indicating high fidelity.

**TABLE 4.** Fidelity of THRIVE intervention product availability by location: other healthy foods and beverages^1^

	Nation A				Nation B			
	Intervention store 1, % (*n* = 28)	Intervention store 2, % (*n* = 22)	Control store 1, % (*n* = 21)	Control store 2, % (*n* = 21)	Intervention store 3, % (*n* = 20)	Intervention store 4, % (*n* = 23)	Control store 3, % (*n* = 17)	Control store 4, % (*n* = 17)
Reach-in food cooler								
Canned tuna/tuna kit	96^2^	100^2^	0	0	100	100	12	0
Canned chicken/chicken kit	96^2^	100^2^	100^2^	0	100	86	0	0
Cheese sticks	96	9^3^	0	0	90	100	50	0
Yogurt	89	14	0	14	98	87	6	0
Hard-boiled eggs	^4^	^4^	^4^	^4^	^4^	100	47	0
Sandwiches	^4^	^4^	^4^	^4^	90	87	0	0
Wraps	^4^	^4^	^4^	^4^	89	84	0	0
Hummus packs	^4^	^4^	^4^	^4^	83	89	6	0
Bottled water	^5^	^5^	^5^	^5^	100	100	29	0
Milk/milk substitute	^4^	^4^	^4^	^4^	81	91	22	0
Endcaps								
Nutrition bars	92	86	5	25	94	81	2	0
Jerky	100	97	89	79	86	100	3	0
Nuts	97	93	93	24	99	97	0	0
Trail mix	63	56	57	18	80	78	3	0
Pickles	^4^	^4^	^4^	^4^	100	100	0	0
Cereal and oatmeal stand								
Oatmeal kits	97	95	0	70	^4^	^4^	^4^	^4^
Cold cereal	96	89	0	3	100^6^	100^6^	0	0
Beverage cooler								
Bottled water	100	100	100	100	100	100	12	6
All other healthy foods and beverages^7^	93	76	40	30	93	93	11	<1

1 THRIVE, Tribal Health Resilience in Vulnerable Environments.

2 These items were located in the grocery shelves in these stores.

3 This item was also located in the deli express cooler, which was not a location that was evaluated.

4 These items were not intervention items for this Nation; hence, these items were not evaluated in these stores.

5 This item is an intervention item for this Nation, but it was located only in the beverage cooler.

6 These items were located on the endcaps in these stores.

7 Combined fidelity of all other healthy foods and beverages in reach-in food cooler, endcaps, cereal and oatmeal stand, and beverage cooler.

### Implementation in control stores

#### Promotional materials

None of the control stores in either Nation used intervention promotional materials.

#### Fruits and vegetables

Due to consumer requests, some control stores sold some THRIVE intervention items during the intervention period. The overall availability of all intervention food and beverage items (fruits, vegetables, other healthy foods, and beverages) ranged from 0.2% to 45% (median: 31.5%) in control stores in both Nations. The overall availability of fruits and vegetables in control stores ranged from 0% to 52% (median: 47.5%) (Table 3). Fresh fruit availability in control stores ranged from 30% to 100% (median: 85.5%) in Nation A and from 0% to 82% (median: 17.5%) in Nation B during the intervention periods. One control store in Nation B did not have any fresh fruit availability, whereas the other control store had some fresh fruit available in up to 82% of the evaluations. Canned fruit availability ranged from 0% to 46% (median: 0%) in Nation A. One control store in Nation A did not have any canned fruit availability, whereas the other control store had some canned fruit available in up to 46% of the evaluations. Canned fruit was not listed as an intervention item in Nation B; therefore, it was not evaluated. The availability of packaged fresh vegetables in control stores was low in both Nations (Nation A: 0–22%; Nation B: 0–35%), and 100% vegetable juice availability varied more in Nation A control stores (17–100%; median: 58.5%) and was low in Nation B control stores (0–17%). The availability of salads was also low in Nation A control stores (22–34%), and these were not carried in Nation B control stores.

#### Other healthy foods and beverages

The availability of other healthy foods and beverages varied between the control stores in the 2 Nations. The overall availability of other healthy foods and beverages in control stores ranged from 0.4% to 40% (median: 20.5%) (Table 4). Control store 1 in Nation A carried canned chicken 100% of the time; however, neither control store in Nation A carried canned tuna. Availability of canned tuna and canned chicken was low in Nation B, ranging from 0% to –12%. Control stores in both Nations carried cheese sticks 0% of the time, except for control store 3 in Nation B (50%). The availability of yogurt ranged from 0% to 14% in all 4 control stores. Snack availability (nutrition bars, jerky, nuts, and trail mix) ranged from 5% to 93% (median: 41%) in Nation A and from 0% to 3% in Nation B. Availability of bottled water was 100% in Nation A and ranged from 6% to 29% (median: 17.5%) in Nation B (including reach-in cooler and beverage cooler locations).

## Discussion

Interventions that increase access to healthy foods are key to addressing disproportionate rates of obesity and other diet-related health disparities in communities with poor food environments (7), including rural AI communities (14). To our knowledge, the THRIVE study is the first healthy retail intervention in tribally owned convenience stores with a detailed process evaluation tool. The tool, developed by tribal–university study staff, assessed the availability, quantity, placement, and promotion of targeted intervention items and was tailored to fit the layout and ordering processes of each store.

Similar to a previous study (10), we found that promotional materials were displayed with high fidelity in the majority of intervention stores in both Nations. These findings are related to study results from the individual-level THRIVE data, which showed that Nation A participants who frequently shopped at the intervention stores noticed promotional materials on endcaps, reach-in coolers, and grocery shelves, and Nation B participants noticed food displays on the endcaps, reach-in coolers, and grocery aisles (18). Low fidelity of some promotional materials was due to management using the promotional signage on other healthy food items that were not evaluated in the process evaluation tool.

Overall, the THRIVE study was implemented with moderate to high fidelity, similar to other food retail interventions (21, 23–25). A previous study addressed the need for future interventions to include the promotion of healthy beverages as well as foods (10), which was implemented in the THRIVE intervention.

Although individual-level findings from THRIVE indicate that fruit and vegetable intake did not increase during the intervention period, the intervention did increase both availability and purchasing of fruits, vegetables, and other healthy foods in convenience stores in both Tribal Nations (18). Shopping frequency was also related to healthy food purchases from the reach-in food cooler and grocery aisle foods marked with promotional signage (18). Furthermore, individual-level findings showed that compared with preintervention, participants in both Tribal Nations perceived an increase in postintervention placement and promotion that encouraged the purchase of healthy food and beverage items (18). In addition to previously reported individual-level results, previously reported store-level results indicate that intervention store NEMS scores increased from baseline to follow-up in 6 food categories (19). All of these individual- and store-level results endorse the conclusion that intervention components were implemented with moderate to high fidelity and increased healthier food and beverage availability as well as encouraged purchasing of healthier food and beverage items.

To increase fruit and vegetable intake, Tribal Nations should consider implementing healthy retail interventions in all tribally owned and affiliated operations, in addition to convenience stores. Since the beginning of the THRIVE intervention, the Choctaw Nation of Oklahoma has taken strides to accomplish this with the opening of its healthy café, Roots, located on the new tribal headquarters campus in Durant, Oklahoma. The new café offers healthy meals at a discounted rate to tribal citizens and employees. Chickasaw Nation has taken a similar approach in its hospital cafeterias, including the Okchamali's Café, located in the Chickasaw Nation Medical Center in Ada, Oklahoma. These new cafés honor Chickasaw Nation's commitment to healthy eating by working together with the Nation's registered dietitians to ensure that offerings are appropriately portioned and nutritious. To continue the movement of revitalizing the food environment for Native people, tribal leadership should consider adopting similar approaches as those discussed previously and also intervene with other tribally owned and operated facilities that serve food, such as schools, grocery stores (9), nutrition assistance programs, and elder programs.

This study has some limitations. Gaps exist between process evaluations in 1 of the Tribal Nations due to the inconsistency of internet connection across the various rural stores. Although study staff tried to prepare for this issue by having backup paper versions of the process evaluation tool on hand, on several occasions staff thought they had a connection to the internet but portions of the survey were not successfully uploaded, evident only later to university research staff once the survey documents were downloaded and reviewed. For future process evaluations administered in rural areas, a staff member should be available to do “live checks” while the person conducting the process evaluation is in the field to ensure that the data are being saved to the server. Another cause for gaps in process evaluations was oversight staff turnover. The university was training a new staff member to oversee this aspect of the study, and the training process delayed the rapid reporting of this issue to tribal staff for several weeks. In addition, the process evaluation schedule in 1 store was delayed by 1 mo due to additional time needed to set up the intervention layout in that store.

Another limitation is contamination in control stores. Packaged intervention snacks were introduced in control stores halfway through the intervention. This occurred as a result of ordering processes as well as consumer demand for the specific intervention foods that were available in other stores but not in their own stores. Third, having 2 tailored versions of the tool made it difficult to report aggregate fidelity. Furthermore, some other positive changes within the stores that went above and beyond specific intervention strategies and improved the food environment were not captured by our tool. Another limitation was that our process evaluation tool did not capture any qualitative data regarding the implementation process from store owners. Future process evaluations should capture this information to provide more context about the implementation process for healthy retail interventions. Future process evaluations should also include more qualitative variables to capture other positive environmental changes that are not specific to the intervention, such as using promotional materials on other healthier items and increased availability of other healthier items within the stores. Last, due to changes in vendor availability, some substituted intervention items were not captured on the process evaluation tool, which may explain some reported lower fidelity of certain items. Similar limitations are reported in a previous study (10).

In conclusion, the THRIVE intervention was implemented with moderate to high fidelity in the intervention stores in both Tribal Nations. Our results suggest that it is feasible for healthy retail interventions to increase availability and promote healthy foods in tribally owned convenience stores. The process evaluation tool we used can be adapted to other food environment interventions and can be used electronically. Although our tool proved useful in assessing the fidelity of the intervention, some qualitative variables could be included on future process evaluations to capture additional information related to positive environmental changes that are not specific to the intervention. Furthermore, although we were able to obtain and extract weekly sales data for all intervention items, future tools should possibly include price data of intervention items if weekly sales data cannot be captured. Future research using detailed process evaluations in other tribally owned convenience stores is needed to validate the tool and to determine best practices for future food environment improvement studies.

## Supplementary Material

nzz073_Supplemental_Tables

## Notes

Author disclosures: TET, ALS, MBW, CVL, CJN, TKC, J Standridge, JF, J Spiegel, JO, MG, AW, and VBBJ, no conflicts of interest.

Supplemental Tables 1 and 2 are available from the “Supplementary data” link in the online posting of the article and from the same link in the online table of contents at https://academic.oup.com/cdn/.

This article appears as part of the supplement “Proceedings of the Third Annual Conference on Native American Nutrition,“ sponsored by the Shakopee Mdewakanton Sioux Community’s Seeds of Native Health campaign through a gift to the University of Minnesota. The guest editors of the supplement, Treena Delormier and Mindy S Kurzer, have no conflicts of interest. The opinions expressed in this publication are those of the authors and are not attributable to the sponsors or the publisher, Editor, or Editorial Board of *Current Developments in Nutrition*. Publication costs for this supplement were defrayed in part by the payment of page charges.

Abbreviations used: AI, American Indian; CBPR, community-based participatory research; IRB, institutional review board; NEMS-S, Nutrition Environment Measurement Survey in Stores; THRIVE, Tribal Health Resilience in Vulnerable Environments.
