# Supplementary material for: Process Evaluation Tool Development and Fidelity of Healthy Retail Interventions in American Indian Tribally Owned Convenience Stores: the Tribal Health Resilience in Vulnerable Environments (THRIVE) Study
Source: Curr Dev Nutr. 2019 Jun 25;4(Suppl 1):33–41. doi: 10.1093/cdn/nzz073 (PMC7101486; doi:10.1093/cdn/nzz073)
Supplement: nzz073_Supplemental_Tables [file nzz073_supplemental_tables.docx]

**Supplemental Table 1.** Number of THRIVE Promotional Materials Collapsed for Each Category in Intervention Stores

|  | **Nation A** | | **Nation B** | |
| --- | --- | --- | --- | --- |
|  | **Intervention Store 1** | **Intervention Store 2** | **Intervention Store 3** | **Intervention Store 4** |
|  | **N** | **N** | **N** | **N** |
| **Reach-in food cooler** | 1 | 1 | 2 | 1 |
| **Endcaps** | 1 | 1 | 2 | 2 |
| **Grocery shelf** |  |  |  |  |
| Whole wheat bread | 1 | 1 | 0^a^ | 0^a^ |
| Canned goods | 1 | 1 | 1 | 1 |
| Nuts/trail mix | 1 | 1 | 1 | 0^b^ |
| Nutrition bars | 1 | 1 | 1 | 1 |
| Low-fat/baked chips | 0^a^ | 0^a^ | 1 | 1 |
| Pickled quail eggs | 0^a^ | 0^a^ | 0^a^ | 1 |
| Beef jerky | 0^b^ | 0^b^ | 1 | 0^b^ |
| **Fruit basket** | 0^b^ | 0^b^ | 2 | 2 |
| **Beverage cooler** | 2 | 2 | 2 | 2 |
| **Beverage fountain** | 0^a^ | 0^a^ | 1 | 1 |
| **Gas pump** | 0^a^ | 0^a^ | 1 | 1 |

^a^Items were not listed as intervention items in this store.

^b^Items were listed as intervention items, but were not promoted in this store.

**Supplemental Table 2.** Number of THRIVE Intervention Products Collapsed for Each Category in Intervention and Control Stores

|  | **Nation A** | **Nation B** |
| --- | --- | --- |
|  | **N** | **N** |
| **Reach-in food cooler** |  |  |
| Fresh fruit |  |  |
| Sliced apples with caramel/peanut butter | 0^a^ | 2 |
| Fruit and yogurt parfait | 1 | 1 |
| Fresh fruit cups | 1 | 2-4^d^ |
| Canned fruit |  |  |
| Orange fruit cups | 1 | 0^a^ |
| Peach fruit cups | 1^c^ | 0^a^ |
| Mixed/tropical fruit cups | 2^c^ | 0^a^ |
| Fresh vegetables |  |  |
| Vegetable packs | 1 | 1 |
| 100% vegetable juice | 2 | 4 |
| Salads | 3 | 4 |
| Canned tuna/tuna kit | 1^b^ | 1 |
| Canned chicken/chicken kit | 1^b^ | 1 |
| Cheese sticks | 1 | 1 |
| Yogurt | 1 | 1-2^d^ |
| Hard-boiled eggs | 0^a^ | 1 |
| Sandwiches | 0^a^ | 3 |
| Wraps | 0^a^ | 4-5^d^ |
| Hummus packs | 0^a^ | 2 |
| Milk/milk substitute | 0^a^ | 3-4^d^ |
| **Endcaps** |  |  |
| Nutrition bars | 11 | 9 |
| Jerky | 3 | 3 |
| Nuts | 7-8^d^ | 7 |
| Trail mix | 4 | 2 |
| Pickles | 0^a^ | 1 |
| **Fruit basket** |  |  |
| Apples | 1 | 2 |
| Oranges | 1 | 1 |
| Bananas | 1 | 1 |
| **Cereal and oatmeal stand** |  |  |
| Oatmeal kits | 3 | 0^a^ |
| Cold cereal | 2 | 1 |
| **Beverage cooler** |  |  |
| Bottled water | 1 | 1^e^ |

^a^These items were not intervention items for this Nation; hence, these items were not evaluated in these stores.

^b^Intervention items were located in the grocery shelves for this Nation.

^c^Intervention items were located in the grocery shelves for one intervention store and one control store in this Nation.

^d^The number of items differed in the two intervention stores; therefore, a range of items is provided.

^e^This item was also located in the reach-in cooler for this Nation.
